# The value of work: Addressing the future of work through the lens of solidarity

**DOI:** 10.1111/bioe.12507

**Published:** 2018-09-18

**Authors:** Barbara Prainsack, Alena Buyx

**Affiliations:** ^1^ Department of Political Science University of Vienna Austria Europe; ^2^ Department of Global Health and Social Medicine King’s College London United Kingdom; ^3^ Institute of History and Ethics in Medicine Technical University Munich Germany

**Keywords:** automation, digitization, employment, future of work, labour, solidarity, work

## Abstract

Designing the future of work is crucial to the health and well‐being of people and societies. Experts predict that developments such as the advancement of digital technologies, automation, and the movement of manufacturing jobs to low‐wage countries will lead to major transformations in the labour market, and some foresee significant job losses. Due to the close relationship between employment and health, major job losses would have significant negative impacts on the health and well‐being of individuals and societies. Job losses would also pose a major challenge to solidaristic support within societies because they would negatively affect the recognition of similarities among people, which is vital for solidaristic practice and institutions. To prevent these negative effects, a fundamental redesign of the relationship between work and income is necessary. And for this project to succeed, we need to reconsider of the value of work. Building on definitions of flourishing people and societies, we argue that the value of work should not be determined by the labour market, but according to its importance for society. Using a solidarity‐based framework we argue that such a re‐valuation of work will help to ensure social cohesion and increase reciprocity in our societies. It will serve as a foundation upon which we can reconfigure the relationship between work and income without risking the loss of social cohesion and solidarity.

## 1 THE FUTURE OF WORK – WHAT IS AT STAKE?

In recent years, a lot of attention has been paid to the relationship between inequality and health, finding that across countries, societies with fewer inequalities do better on a range of outcomes including health,^1^ and within countries, people with higher socioeconomic status tend to have better health outcomes.^2^ An important element within this well‐established dynamic is the relationship between paid employment and health. Several decades of research have shown that paid employment positively contributes to people’s well‐being and health, and vice versa.^3^ Psychological, economic, and social factors play a role in this. In many societies, people’s income – and thus their ability to afford good housing in healthy environments, good food, and high‐quality healthcare – is directly or indirectly dependent on paid employment.^4^ On a macro level, there is a strong association between labour market inequality and unfavourable population health outcomes.^5^ At the individual level, job insecurity, marginal or informal employment, loss of employment and long‐term unemployment all impact negatively on health and well‐being.^6^ Conversely, there is a strong correlation between high levels of stable employment in a given society and better individual and public health.^7^

In addition to the effects on health, in Western societies, work and employment have also long been recognized as some of the most important conveyers of social standing and status. Wages, social capital and all the other trappings of work and employment demarcate social status, power and access to resources in the modern world. The jobs people have shape and structure daily lives on an individual, social and communal level. Work and employment also have significant impacts on how individuals perceive themselves within the fabric of their communities and societies and how they, in turn, are regarded by others.^8^ This directly impacts on people’s feelings of worth, confidence and contentment.^9^ Being in paid employment also broadens people’s options for participation in our societies beyond economic factors (such as disposable income). Indeed, change in, or loss of, steady employment are regarded as a major life events, and many of the negative health effects of (long‐term) unemployment or unstable/precarious forms of employment come about through psychological and social pathways that accompany changes in social and financial status, loss of social recognition and dignity etc.^10^ Those who lose their job often report feeling that they have not only suffered financial losses, but also loss of a sense of belonging, meaning, and of purpose.^11^ While these consequences are felt across the whole socioeconomic, professional gradient, they have the biggest negative impact in exactly those low‐skilled groups that are likely to be most affected by the future transformations of work and labour.^12^

Importantly, paid employment is also one of the determinants of social cohesion in Western societies.^13^ Loss of paid employment, particularly long‐term and at a larger scale, has destabilizing and fracturing effects on communities.^14^ Employment, or the lack of it, not only affects how people view themselves and their place and participation in community and society, it also influences how people see others and whether they consider others as similar to themselves, and worthy of support. If income from paid work remains the main source of income for most people, and if growing parts of the population are no longer able to find paid work (that pays enough for them to make a living), then this would not only amount to a threat to the health and dignity of the unemployed. In addition, those without employment could also be regarded as permanently ‘different’ – as is already happening in the case of the long‐term unemployed and the underemployed.^15^ Significant changes to the ideal of full paid employment without adequate replacement for those unable to find it could mean that unemployment and unpaid work would be even greater structural features of our societies than is the case today – with the problematic effects on health and social cohesion described above.

### 1.1 No work or new work? Optimistic and pessimistic scenarios

These considerations are of particular importance in the context of the so‐called future of work (FOW) challenge.^16^ This is the challenge stemming from technology actually or potentially replacing large areas of human work, and the subsequent transformation of societies. The advancement of digital technologies and computation alone, mostly in the format of artificial intelligence (AI) and robots, is expected by some authors to lead to significant job losses around the globe.^17^ The proportions of predicted job losses vary significantly according to the different assumptions that modellers make, but virtually everyone agrees that there will be dramatic shifts in employment patterns.^18^ While a wide range of scholarship is now debating the effects of these shifts on national economies, their consequences for individual and public health, given the noted importance of paid employment for positive health outcomes, have been a relatively neglected issue. Similarly, little attention has been paid to how these shifts may affect social cohesion. If we leave these issues unattended, then these developments are likely to lead to ever‐more fractured, divided societies.^19^

Not all expectations and predictions of the FOW are pessimistic. Some authors believe that that job losses due to automation will be compensated, at least partly, by new work: first, by jobs that are created to meet newly emerging needs and practices in society, such as jobs in retailing; second, some experts argue that automation itself will create new jobs.^20^ They cite historical examples for this very mechanism such as the increase of available jobs in accounting due to a drop in the cost of bookkeeping brought about by electronic spreadsheets.^21^

Although it is impossible to predict how many new jobs this will create, it is plausible to expect that automation will generate the need for human oversight. In the 1980s, Lisanne Bainbridge argued that automated systems can inevitably only be designed for some, and not all possible scenarios. Notwithstanding developments in machine learning, this still applies for the foreseeable future: ‘the more advanced a control system is, so the more crucial may be the contribution of the human operator’.^22^ Bainbridge’s observations in the 1980s foreshadowed the notion of complementarity between humans and machines that is part of today’s debates of, for example, ‘smart industries’ characterized by interoperability of machines and people.

Labour market experts predict that there will be new (self‐)employment opportunities, especially for adaptable and creative people with good interpersonal skills.^23^ But those who have none or few such skills that are considered valuable on the human labour market might be left jobless. Moreover, even if society will have new needs that are best met by human work, and if automation requires human oversight, it is not a given that these tasks will be done by people who get paid enough to make a living. Factors such as the (renewed) increase of the proportion of income coming from capital compared to work and labour,^24^ policies that have allowed that wages stop tracking productivity growth,^25^ and the increase of atypical and precarious employment formats^26^ have led to a situation where many working people can no longer make a living from what they earn through their labour. The problem of the working poor will not be solved by the creation of new jobs. Dealing successfully with the FOW challenge requires much more than a strategy of how to deal with automation; it requires a reordering of our societies, at the core of which must be a reconsideration of the value of work.

In the following, we lay out an argument detailing that we should take the FOW challenge as an opportunity to ‘reset’ the organization of our societies in preparation, to create circumstances that are conducive to greater equality, health, social cohesion, and the flourishing of people and communities. We use the term ‘flourishing’ in a twofold manner. At the level of people, we understand flourishing to ‘require doing or being well in the following five broad domains of human life: (i) happiness and life satisfaction; (ii) health, both mental and physical; (iii) meaning and purpose; (iv) character and virtue; and (v) close social relationships’.^27^ At the level of societies, flourishing societies are those that ensure that dignified living circumstances are available to everybody. We believe that social cohesion is an emergent property of flourishing societies.^28^

We sketch a few important measures to take us on this road.

## 2 SOLIDARITY AND THE FUTURE OF WORK

A large a number of policy reports have suggested a variety of ways to address the FOW challenge.^29^ There is broad consensus that a strategic response is needed to mitigate the effects of changes in the labour market, and that doing so is complex. There is less accord regarding the directions that societies should take in tackling this challenge. This disagreement centres on two key areas; first, an appropriate strategy to be adopted in response to the challenge, and second, the fundamental normative tenets that future societies should be built upon. It is this latter aspect that is the specific focus of this article.

Our key point is that to work towards flourishing societies and preserve social cohesion, we must first ensure the continued existence of the preconditions for social cohesion. We propose that this can be achieved by changing how we value, and also how we define, work. Put differently, we argue that solidarity can help us to shape the circumstances that enable flourishing societies against the backdrop of changes to the way we work, and at the same time increase social cohesion and reciprocity.

In our recent book^30^ we propose a definition of solidarity to enhance the analytical value of the concept, and enable us to distinguish it clearly from related terms and phenomena. Building upon a long tradition of scholarship on solidarity, we define solidarity as ‘enacted commitments to accept costs to assist others with whom a person or persons recognise similarity in a relevant respect’.^31^ Our definition deviates from many previous understandings of solidarity in two main ways. First, we consider solidarity not merely a value or a principle that would be nice to have, but a personal and social practice.^32^ Analysing or designing circumstances to support solidaristic practice or policy thus always requires careful attention to and analysis of the relevant context in which the practice occurs. Solidaristic practice can happen at any social level or ‘tier’, ranging from transient solidaristic interactions between individual people, to more institutionalized group solidarity practices, to solidaristic arrangements in the form of legal, contractual, or administrative norms and rules (e.g. universal health insurance, progressive taxation, etc.).^33^

Second, our understanding of solidarity is anchored in a relational concept of personhood – that is, on conceiving of individuals as embedded in, open to and dependent on their social, natural and artefactual environments.^34^ Such a relational understanding of personhood implies that people’s subjectivities and also their interests are influenced by their relations to others, and that, hence, we cannot distinguish neatly between self‐interested and other‐directed action. It flows naturally from this understanding of the person that most people want to engage in other‐directed, prosocial practice that helps them and others to flourish. The key about solidarity – and one of the aspects that sets solidarity apart from notions such as altruism – is that people enact solidarity with others with whom they consider themselves *connected* in some sense. The characteristics that give rise to this are not necessarily ‘objective’ similarities, such as belonging to the same faith or religion or having gone to the same school: they are whatever a person, groups or entire societies, deem relevant in a specific practical situation. ‘Similarities in a relevant respect’ that give rise to solidaristic practice can thus be a fleeting feature of a situation, such as the bonding over a shared musical experience or missed flight, or they can be something that lies at the core of a person’s or group’s identity, such as a religious faith, a commitment to specific values, or suffering from a specific illness. These commonalities are the ‘motivational trigger’ for solidaristic practice and, as such, they need to matter in a specific situation or for a specific context, despite the simultaneous existence of differences among people and groups.^35^ In other words, societies where solidarity flourishes need not be homogeneous or lacking diversity. People might indeed be very different in many respects from people they act in solidarity with, or from those they are united with through solidaristic policies and laws. At the same time, however, people share similarities with others that can give rise to action in specific contexts. We will return to this important aspect of solidarity below.

Importantly, solidaristic practice between individuals, particularly of the transient kind, can occur without any expectation of reciprocity; indeed, if reciprocity is the sole or the decisive reason for a practice it cannot be classified as solidaristic.^36^ However, for solidarity to solidify and grow at a systemic level, a certain level of reciprocity is needed. That is, institutionalized solidarity practices, such as laws mandating tax or other financial contributions towards, say, a publicly funded healthcare system or unemployment benefits, will be more stable and effective if those who contribute can, in turn, expect to receive support if and when they need it, even if it is support of another kind (indirect reciprocity).^37^ Further, overall attitudes of reciprocity in a society will help maintain and enliven solidaristic arrangements, a point we return to below.^38^

We argue that societies that support and facilitate practices of people who (a) recognize relevant similarities with many others, and (b) support others on the basis of this recognition, are those with greater social cohesion and greater individual and societal well‐being. Beyond that, there are certain societal features that facilitate solidaristic practices and institutions, such as economic and political stability, trustworthy institutions that respect the rule of law, and laws and bureaucratic and administrative norms which are transparent and accountable.

## 3 HOW THE FUTURE OF WORK CHALLENGE AFFECTS SOLIDARITY

Taking a solidarity‐based perspective throws into sharp relief two major challenges the transformation of work would bring, if the changes do indeed occur in the way many currently expect. Arguably the most important of these is the effect that major job losses could have on the recognition of *similarity* with others, which is vital for any solidaristic practice. Early sociologists, such as Emile Durkheim, have already highlighted the close connection between changing forms of labour and social cohesion. Durkheim (1893)^39^ considered the growing division of labour the root cause for changing forms of solidarity. He argued that people in pre‐modern societies, prior to the division of labour, were bound together by so many commonalities – namely in the way they laboured, worked and lived – that cohesion and cooperation were almost ‘automatic’; people cooperated like the different parts of a well‐oiled machine. Durkheim called this ‘mechanical solidarity’. With the increasing division of labour, people still depended on each other and cooperated, but in different ways. Durkheim used the term of ‘organic’ solidarity to describe social cohesion that is characterized by a kind of interdependence that is not primordial but functional – people co‐operate because, if they did not, they would not be able to fulfil their specific functions. Like the organs in the body, the functions of each one are different, but they depend on each other to do their work.

The kind of ‘organic’ solidarity that, according to Durkheim, characterizes modern societies, is less self‐evident and weaker than the ‘mechanical’ solidarity prevalent in pre‐modern societies. This is arguably the case because of the practices and forms of othering brought about by the division of labour. As a result of the different, and potentially competing, interests of members of different professional groups (factory workers have different interests from farmers or bankers for instance, in many respects), and because of the physical, functional and status separations between them, there are fewer features that can give rise to the mutual recognition of similarities and commonalities among people, which are, as we noted, the ‘trigger’ for solidaristic practice. Further, the new mobility patterns and a greater diversity in terms of culture, nationality, religion and language in contemporary societies have increased the number of divisions between different groups and categories of people.

This does not mean, however, that the dissolution of solidarity is inevitable in diverse societies. The lines that separate different groups of people are not necessarily obstacles for cohesion and mutual support. We argued above that solidarity depends on people recognizing similarity in a relevant respect with others, and making these similarities the foundation of action – instead of the many ways in which we are different from others. As noted above, the recognition of similarity that underpins solidaristic practice is not merely a matter of acknowledging ‘objectively’ existing similarities. Instead, the process of recognizing similarities in a relevant respect is a deeply personal, social and political process.^40^ We recognize in others characteristics that are meaningful to us, and they are meaningful to us because we have been taught that these categories matter (e.g., being a citizen of the same nation, of the same gender, belonging to the same religion etc.), or we have experienced that they are important (e.g., being a cancer patient, being a woman). In this sense, the recognition of similarity is a ‘subjective’ matter, but it is by no means arbitrary, and certainly not merely individual. Recognizing similarity is often a shared practice influenced by the categories and metrics that support political and economic goals. The increasing stigmatization of the unemployed – which starts with a disgruntlement over ‘our’ tax money going to support ‘them’, and ends with hatred and violence towards ‘benefit scroungers’ – is made possible by the existence of political and social categories that allow us to separate between those who are in employment and those who are not. And, as described above, because employment is so closely associated with both income and social status in our societies, it also has a strong relationship with how much people are seen as contributing to society. Those who are not in employment, if they are at an age where they could be, are thus increasingly seen as ‘free riders’, as not contributing their share to society.^41^

This perspective hinders social cohesion, because it is inherently divisive. Given the scale and depth of transformation that work and employment are undergoing at the moment, it is to be expected that large proportions of the population will be ‘othered’ and labelled as non‐contributors. And so, on top of the detrimental health effects from decreasing employment, social cohesion and the willingness to support each other would probably also decrease in future societies, leaving them divided and fractured, and without the conditions to flourish and prosper. Therefore, in order to defend and enhance solidarity and social cohesion as we shape the future of work and the future of our societies, shared understandings of the value and importance of work, and the range of ways in which people are seen to contribute to society need to change. If we want to prevent growing societal divisions that will lead to a deterioration of well‐being at the individual and population level, we need to create societies in which everybody who contributes something valuable to the functioning of society is seen to be ‘working’. 42 This, we argue, will create a foundation upon which further debates and decisions on how we reconfigure the relationship between employment and income can safely take place, without risking that groups that are considered of no value on the labour market (as it is conceived of currently) are increasingly marginalized. Because it is likely that the size of these groups will grow in the near future, and because we envisage that they will include those who are already marginalized and vulnerable regarding their health and well‐being, such a re‐valuation of work and labour is a highly pressing issue.

In the following section we sketch three steps that are necessary to reach this goal of a re‐valuation of work.

## 4 WAYS FORWARD: HOW SOLIDARITY CAN HELP. CONCEPTS AND PRACTICE

### 4.1 A new approach to the value of work: Does it contribute to the basic functioning of societies?

A significant proportion of work which is key to the functioning of our societies is currently unpaid.^43^ ‘Homemaking’, the work of raising and educating children, caring for the elderly, and a lot of voluntary social, artistic and community work are but a few examples.^44^ As Kate Raworth argued, ‘... mainstream economic theory is obsessed with the productivity of waged labour while skipping right over the unpaid work that makes it all possible’.^45^ Drawing upon Neva Goodwin, Raworth argues that the unpaid work that people – mostly women – are doing to care for children or the elderly, for example, is not marginal at all but it represents the ‘core economy’. It could be argued that such currently unpaid work does indeed represent the core economy because it includes tasks that ensure that people’s fundamental needs are met; the latter being health, shelter, food, education and meaningful relations to other people.^46^

In order to preserve the solidarity that is vital for the flourishing and social cohesion in future societies, we must overcome the prejudices that lead us to attribute more value to jobs that fare better at the labour market than to the (often unpaid or underpaid) work necessary for the basic functioning of our societies. The key questions for the re‐valuation of different types of work in our society should be: what are ‘resources that comprise and sustain social life’?^47^ What are the human practices that comprise and sustain these resources? We must recognize that many forms of underpaid or unpaid work are at least as important for society as jobs that are highly remunerated. Currently, tasks that merely increase financial profits of a small group of individuals are among the best paid in our societies; for the basic functioning of our societies they have very little or even negative value (e.g., the creation of risky financial instruments).^48^ Grounded in this recognition, we must frame our perspective in a new understanding of the value of different kinds of work, and the different kinds of resources and social contributions that each kind creates (artistic, financial, social, familial, etc.). Doing so will allow us to recognize and reward those people involved in a far broader range of work than the current paradigm.^49^ In turn, this recognition can serve as the basis for wider societal solidarity, and reverse some of the splintering of social cohesion driven by changes to employment status. An unpaid person providing part‐time care for an ailing relative would no longer be an unemployed ‘skiver’, but would be seen as – and compensated for – carrying out crucially important work. Likewise, the contributions of a part‐time barista who spends several hours per week to help elementary school children with their reading skills would be recognized beyond her minimum‐wage job. She would receive remuneration for this work as a recognition of its value for society.

### 4.2 Compensation for valuable work

Remuneration for work that is of value for functioning societies is crucial in this model. But how should it be decided, and by whom, what work is of value, and how it should be remunerated? We suggest the creation of a statutory body comprising professional experts from a variety of disciplinary and practical backgrounds, as well as citizens who do a range of paid and unpaid work. This body would be tasked with developing criteria to assess the societal value of work; we propose that public bodies make reference to work based on a capabilities approach of human flourishing when developing these criteria.^50^ The level of societal value of specific work, in combination with other factors including the complexity, demands, and responsibilities coming with these tasks, as well as the training, education, and other investments that people have made to obtain the skills necessary to do the task, would translate into a novel scale of remuneration.

Public investments would need to be made to compensate people who do these tasks according to the point on the scale for each task. Such compensations could take the form of payments to those who carry out (currently unpaid) caring work, or they could top‐up salaries and wages received from paid employment if the societal value of the task exceeds what people earn.^51^ Tasks that have little value for the flourishing of societies – such as investment banking or telemarketing – would continue to exist if remunerated by private employers. We would expect, however, that a shift in the criteria according to which we remunerate work would also lead to a stigmatization of tasks that do not create any societal value.

Developing such a form of alternative remuneration will require participation from a wide range of societal actors so as not to cement existing biases. However, we believe that the current system of remuneration of work will no longer be suitable for societies that are undergoing the dramatic changes in employment patterns described earlier. We believe that any alternative model will have to consider the thorny task of determining more specifically, and more substantially, what we value as a society (e.g., when determining a novel scale of compensation). Developing and implementing a model for the remuneration of work according to its societal value would not only lead to increased and systematic financial recognition of so far unpaid volunteering or caring work. It would also mean that we are moving away from establishing the value and remuneration of work predominantly by supply and demand at the job marketplace, to a system where the value and remuneration correspond to the importance, broadly understood,^52^ of the work or labour to the flourishing of societies.

Alternatively, societies could decide to operate on the presumption that virtually everybody makes a contribution to society in one way or another, and implement unconditional/universal basic income (UBI) as a ‘lump sum’ compensation for people’s contributions. Also here, the re‐valuation of work according to the role it plays in enabling flourishing societies would have an effect: it would mean that the justification of UBI would not be one of welfare (leaving UBI open to the accusation of ‘paying people for doing nothing’), but one of compensating people for things they are contributing to the flourishing of society.^53^ Finally, another policy option would be the creation of lifetime work and labour accounts,^54^ again based on a novel scale of evaluating work and labour.^55^

What system of remuneration would be most feasible and appropriate, and how to finance it, are very big questions that we will address in another paper. For now, we limit ourselves to pointing out the kinds of broad shifts that need to happen to facilitate the FOW in ways that lead to flourishing societies.

### 4.3 Change of discourse

As discussed above, solidarity‐based approaches pay attention to how similarities between people ‘trigger’ practices of mutual support. Reframing the value associated with different kinds of work in terms of societal value – which, in turn, is dependent on their contribution to the flourishing of societies, rather than how much they are worth on the labour market – offers a way to include those engaged in socially valuable, yet traditionally unrecognized, labour and work within the group of those taken to contribute to society. Consequently, acknowledging the broad social value of atypical and/or unpaid (or underpaid) work minimizes the number of persons who can be defined as social ‘outsiders’ in virtue of the nature of the work that they do. Such recognition and inclusiveness is beneficial for those newly included in the ‘social contributors’ group, because it offers them social validation and recognition of the importance of their contribution to society. It also offers a novel perspective on a range of social issues that may more effectively be addressed through approaches emphasizing solidarity rather than ‘othering’.^56^ If we want flourishing societies, and, in addition, if we want to keep and even enhance solidaristic support even through the coming transformations of work, we need to describe both the problem and the solutions in ways that emphasize what people have in common and not what sets them apart.

## 5 CONCLUSION

The FOW challenge – the expectation of job losses due to the automation of tasks, the movement of low‐skill jobs to low‐wage countries, and other related developments – is considered one of the main challenges that industrialized Western societies are currently facing. Using a solidarity‐based perspective, we have argued that strategies to address this challenge need to start with a reconsideration of the value of work and labour. Such a reconsideration, in turn, should be guided by two steps: changing the way we define valuable work and the implementation of a system that compensates everybody who contributes to the functioning of our societies irrespective of what their contributions are worth on the ‘traditional’ labour market; and a political and social discourse that emphasizes what people have in common, and not what sets them apart. A solidarity‐based perspective highlights the meaning and the implications of the potential tears in the social fabric that are likely to occur, including effects on health and social cohesion. There is little doubt that our societies and our support systems, particularly those organized according to the principle of solidarity, are not prepared for these tears to appear as widely and as rapidly as is currently being envisaged. If we want to avoid fractured societies and a decline in health, well‐being and flourishing, we need to start working towards such transformations now.

## CONFLICT OF INTEREST

The authors declare no conflict of interest.

**Barbara Prainsack** is a professor at the Department of Political Science at the University of Vienna, and at the Department of Global Health & Social Medicine at King’s College London. Her work explores the social, regulatory and ethical dimensions of biomedicine and bioscience. Among other advisory bodies she is a member of the Austrian National Bioethics Committee and the European Group on Ethics in Science and New Technologies advising the European Commission.

**Alena Buyx** is Professor of Ethics in Medicine and Health Technologies and Director of the Institute for History and Ethics in Medicine, Technical University Munich. She works on a wide variety of issues in biomedical, research and public health ethics with a focus on application in practice and policy. Her background is in medicine, philosophy and sociology. She is a member of the German Ethics Council.

## Footnotes

1 Marmot, M. (2015). The health gap: The challenge of an unequal world. London, UK: Bloomsbury.

2 Marmot, M. (2015). *The status syndrome: How social standing affects our health and longevity*. London, UK: Bloomsbury; Wilkinson, R., & Picket, K. (2010). *The spirit level: Why equality is better for anyone*. London, UK: Penguin; Rowlingson, K. (2011). *Does income inequality cause health and social problems*? York, UK: Joseph Rowntree Foundation.

3 World Health Organization (WHO). (2007). *Employment conditions and health inequalities final report to the WHO Commission on Social Determinants of Health (CSDH)*. Retrieved June 27, 2017, from https://www.globalhealthequity.ca/electronic%20library/Employment%20Conditions%20and%20Health%20Inequalities%20Final%20Report%20WHO%20CSDH.pdf

4 Ibid. Marmot, *op. cit.* note 1.

5 WHO, *op. cit.* note 3.

6 For examples, see: Goodman, N. (2015). *The impact of employment on the health status and health care costs of working‐age people with disabilities*. Retrieved June 27, 2017, from http://www.leadcenter.org/system/files/resource/downloadable_version/impact_of_employment_health_status_health_care_costs_0.pdf; Reisine, S., Fifield, J., & Winkelman, D. K. (2016). Employment patterns and their effect on health outcomes among women with rheumatoid arthritis followed for 7 years. *Journal of Rheumatology*, *25*(10), 1908–1916; Harrison, A. S., Sumner, J., McMillan, D., & Doherty, P. (2016). Relationship between employment and mental health outcomes following cardiac rehabilitation: an observational analysis from the National Audit of Cardiac Rehabilitation. *International Journal of Cardiology*, *220*, 851–854; Strully, K. W. (2009). Job loss and health in the U.S. labor market. *Demography*, *46*(2), 221–246. The classical study on this topic is, of course, Jahoda, M., & Zeisel, H. (1974). *The sociography of an unemployed community*. New Brunswick, NJ: Transaction Publishers.

7 Goodman, *op. cit.* note 6.

8 The World Bank. (2013). *World Development Report. Jobs and social cohesion*. Chapter 4, p. 135. Retrieved April 17, 2018, from http://siteresources.worldbank.org/EXTNWDR2013/Resources/8258024‐1320950747192/8260293‐1322665883147/Chapter‐4.pdf

9 Brand, J. E. (2015). The far reaching impact of job loss and unemployment. *Annual Review of Sociology*, *41*, 359–375; Rosen, R. J. (2014, June 9). The mental‐health consequences of unemployment. *The Atlantic*. Retrieved June 27, 2017, from https://www.theatlantic.com/business/archive/2014/06/the‐mental‐health‐consequences‐of‐unemployment/372449/

10 Brand, *op. cit*. note 9; Dekker, S. W., & Schaufeli, W. B. (1995). The effects of job insecurity in psychological health and withdrawal: A longitudinal study. *Australian Psychologist*, *30*, 57–63; Ferrie, J. E, Shipley, M. J., Stansfeld, S. A., & Marmot, M. G. (2002). Effects of chronic job insecurity and change in job security on self‐reported health, minor psychiatric morbidity, psychological measures, and health related behaviours in British civil servants: the Whitehall II Study. *Journal of Epidemiology & Community Health*, *56*, 450–454; Broman, C. L., Hamilton, V. L., & Hoffman, W. S. (1996). The impact of unemployment on families. *Michigan Family Review*, *2*(2), 83–91; Lamont, M. (2000). *The dignity of working men: Mortality and the boundaries of race, class and immigration*. Cambridge, MA: Harvard University Press.

11 Brand, *op. cit.* note 9; Lamont, *op. cit.* note 10; Hochschild, A. R. (2016). *Strangers in their own land: Anger and mourning on the American right*. New York, NY: The New Press.

12 Brand, *op. cit.* note 9.

13 Ibid. Lamont, *op. cit.* note 10.

14 The World Bank, *op. cit*. note 8.

15 Gallie, D. (2004). *Resisting marginalization: Unemployment experience and social policy in the European Union*. Oxford, UK: Oxford University Press; Clasen, J. (2011). *Regulating the risk of unemployment: National adaptations to post‐industrial labour markets in Europe*. Oxford, UK: Oxford University Press.

16 For an accessible introduction see, for example, Avent, R. (2017). *The wealth of humans: Work and its absence in the twenty‐first century*. London, UK: Penguin Random House. For an overview of policy reports see below (note 47);

17 Arntz, M., Gregory, T., & Zierahn, U. (2016). *The risk of automation for jobs in OECD countries: A comparative analysis*. OECD Social, Employment and Migration Working Papers 189, OECD Publishing, Paris. Retrieved Accessed March 27, 2018, from http://www.ifuturo.org/sites/default/files/docs/automation.pdf

18 See also Balliester & Elsheikhi, op. cit. note 16.

19 The World Bank *op. cit*. note 8.

20 Many of these experts are paid by technology companies, e.g. Deloitte (2015). *From brawns to brains: The impact of technology on jobs in the UK*. Retrieved May 6, 2018, from https://www2.deloitte.com/content/dam/Deloitte/uk/Documents/Growth/deloitte‐uk‐insights‐from‐brawns‐to‐brain.pdf; Lomas, N. (2018, Feb 28). AI will create new jobs but skills must shift, say tech giants*. Tech Crunch*. Retrieved May 6, 2018, from https://techcrunch.com/2018/02/28/ai‐will‐create‐new‐jobs‐but‐skills‐must‐shift‐say‐tech‐giants/

21 Levy, F., & Murnane, R. J. (2012). *The new division of labor: How computers are creating the next job market*. Princeton, NJ: Princeton University Press. See also Nedelkoska, L., & Quintini, G. (2018). *Automation, skills use and training, OECD Social, Employment and Migration Working Papers, No. 202*. Paris, France: OECD Publishing. Retrieved April 29, 2018, from http://dx.doi.org/10.1787/2e2f4eea‐en; see also Balliester & Elsheikhi, op. cit. note 16.

22 Bainbridge, L. (1983). Ironies of automation. *Automatica*, *19*(6), 775–779, p. 775.

23 National Academy of Science, Engineering, and Medicine. (2017). *Information technology and the U.S. workforce: Where are we and where do we go from here*? Washington, DC: The National Academies Press, pp. 8–9.

24 Piketty, T. (2014). *Capital in the twenty‐first century*. Cambridge, MA: Belknap; Haldane, A. G. (2015). *Labour’s share. Bank of England Speech. *Trades Union Congress, London, p. 5. Retrieved July 13, 2017, from http://www.bankofengland.co.uk/publications/Documents/speeches/2015/speech864.pdf; Jacobs, M., & Mazzucato, M. (2016). Rethinking capitalism: An introduction. In Jacobs, M. & Mazzucato, M. (Eds.), *Rethinking capitalism: Economics and policy for sustainable and inclusive growth* (pp. 1–27). Chichester, UK: Wiley Blackwell.

25 Berriman, R. (2017). Will robots steal our jobs? The potential impact of automation on the UK and other major economies. *UK Economic Outlook*, p. 1. Retrieved July 13, 2017, from https://www.pwc.co.uk/economic‐services/ukeo/pwcukeo‐section‐4‐automation‐march‐2017‐v2.pdf

26 Kalleberg, A. L. (2008). *Precarious work, insecure workers: Employment relations in transition*. Retrieved July 11, 2017, from http://sawyerseminar.web.unc.edu/files/2011/02/kalleberg_Precarious_Work.pdf

27 VanderWeele T. J. (2017). On the promotion of human flourishing. *Proceedings of the National Academy of Sciences of the United States of America*, *114*(31), 8148–8156, p. 8149.

28 Wilkinson & Picket, *op. cit.* note 2; Raworth, K. (2017). *Doughnut economics: Seven ways to think like a 21st‐century economist*. Chelsea, US: Chelsea Green Publishing, p. 79; Orton, M. (2011). Flourishing lives: The capabilities approach as a framework for new thinking about employment, work and welfare in the 21st century. *Work, Employment and Society*, *25*(2), 352–360; Marks, N., & Shah, H. (2014, September 17). A wellbeing manifesto for a flourishing society. *New Economics Foundation*. Retrieved July 13, 2018, from http://neweconomics.org/2014/09/wellbeing‐manifesto‐flourishing‐society/

29 Recent examples include: International Labour Organization (ILO). (2017). *The future of work. *Retrieved June 27, 2017, from http://www.ilo.org/global/topics/future‐of‐work/lang‐‐en/index.htm; European Political Strategy Centre. (2016). *The future of work. Skills and resilience for a world of change*. Retrieved June 29, 2017, from http://ec.europa.eu/epsc/publications/strategic‐notes/future‐work_en; National Academy of Science, Engineering, and Medicine, *op. cit*. note 23; Baedkel, A. D. (2013, November 27). Future work. Challenges towards 2020. *Scenario Magazine*. Retrieved June 29, 2017, from http://www.scenariomagazine.com/future‐work‐challenges‐towards‐2020/; Breene, K. (2016). What is the future of work? *World Economic Forum*. Retrieved June 29, 2017, from https://www.weforum.org/agenda/2016/01/what‐is‐the‐future‐of‐work/; Nedelkoska & Quintini, *op. cit.* note 21; see also the ‘Future of Work Issue Briefs’ of the International Labour Organization (ILO). Retrieved from http://www.ilo.org/global/topics/future‐of‐work/WCMS_618159/lang‐‐en/index.htm; see also Balliester & Elsheikhi, op. cit. note 16.

30 Prainsack, B., & Buyx, A. (2017). *Solidarity in biomedicine and beyond*. Cambridge, UK: Cambridge University Press.

31 Ibid., p. 43.

32 Ibid., p. 44ff.

33 Ibid., p. 54.

34 Ibid., p. 48ff. See also Taylor, C. (1985). The person. In M. Carrithers, S. Collins, and S. Lukes (Eds), *The category of the person: Anthropology, philosophy, history* (pp. 257–281). Cambridge, UK: Cambridge University Press; Taylor, C. (1989). *Sources of the self: The making of modern identity*. Cambridge, MA: Harvard University Press; Mackenzie, C., & Stoljar, N. (2000). *Relational autonomy: Feminist perspectives on autonomy, agency, and the social self*. Oxford, UK: Oxford University Press.

35 Prainsack & Buyx, *op.cit*. note 30, p. 53.

36 Ibid., p. 63.

37 Ibid., p. 61.

38 There are many other authors writing on solidarity, such as, e.g., those from the special issues on Solidarity in *Bioethics *2012 and 2018. Some of these have critically engaged with our own account (see chapter 3 of our book, *op. cit*, note 30, where we respond to many of the issues raised). In this article, we do not want to focus on specifically our—or, indeed, on others’—solidarity account. Thus, we refrain from discussing conceptual differences between our account and the accounts of other authors. Our concern in this paper is to show what applying a solidarity‐based perspective to the FOW challenge might yield with regards to the implications for future policy and discourse. We do not propose that our own understanding of solidarity is the only one that will yield these benefits.

39 Durkheim, E. (1893). *The division of labour in society*. London, UK: Macmillan.

40 Dean, J. (1996) *Solidarity of strangers: Feminism after identity politics*. Berkeley, CA: University of California Press.

41 For example, Buss, C. (2018). Public opinion towards targeted labour market policies: A vignette study on the perceived deservingness of the unemployed. *Journal of European Social Policy* (in press).

42 42See also: European Group on Ethics in Science and New Technologies. Opinion on the Future of Work. Brussels (forthcoming).

43 Ferrant, G., Pesando, L. M., & Nowacka, K. (2014). Unpaid care work: The mission link in the analysis of gender gaps in labour outcomes. *OECD Development Centre*. Retrieved July 13, 2017, from https://www.oecd.org/dev/development‐gender/Unpaid_care_work.pdf; Levitsky, S. R. (2014). *Caring for our own: Why there is no political demand for new American social welfare rights*. Oxford, UK: Oxford University Press; Cooper, M., & Waldby, C. (2014). *Clinical labor: Tissue donors and research subjects in the global bioeconomy*. Durham, NC: Duke University Press; Grant‐Smith, D., & McDonald, P. (2018). Ubiquitous yet ambiguous: An integrative review of unpaid work. *International Journal of Management Reviews*, *20*, 559–578.

44 From voluntary fire departments to training the local soccer teams, from volunteering in libraries to organizing band festivals, from cleaning up the local park or beach to organizing the social lives of neighbourhoods etc.

45 Raworth, *op. cit.* note 28, p. 79.

46 For the sake of our argument, it is not of central importance how the fundamental needs are justified/defined, whether from a capabilities perspective, or based on some form of fundamental needs theory.

47 Goodwin, N., Nelson, J., Ackerman, F., & Weisskopf, T. (2003). *Microeconomics in context*. New York, NY: Houghton Mifflin.

48 See also Mazzucato, M. (2018)*. The value of everything: Making and taking in the global economy*. London, UK: Penguin Random House.

49 Usually, long‐term employment with a single employer.

50 Martha Nussbaum provided a list of core capabilities in her work. For a society to be at least minimally ‘decent’, it must secure at least a threshold level of these capabilities (as in real opportunities based on individual and social circumstances) to all its citizens. Nussbaum, M. (2011). *Creating capabilities: The human development approach*. Cambridge, MA: Harvard University Press, pp. 10, 30–31. Nussbaum’s list of capabilities has been further refined and clarified in the large literature on the capabilities approach in human flourishing and development.

51 A remaining issue is how those who cannot make a contribution at all (some with disabilities or hereditary illnesses), who no longer can contribute (the elderly, the frail etc.) and those who do not want to contribute would fit into this approach. Systems of unconditional basic income would address this question.

52 Radin, M. J. (1987). Market‐inalienability. *Harvard Law Review*, *100*(8), 1849–1937.

53 The introduction of a basic income should be considered only in conjunction with the provision of basic services including healthcare, transportation, education, and affordable and decent housing. See also UCL Institute for Global Prosperity (2017). *Social prosperity for the future: A proposal for Universal Basic Services*. London: UCL Institute for Global Prosperity. Retrieved April 29, 2018, from https://www.ucl.ac.uk/bartlett/igp/sites/bartlett/files/universal_basic_services_‐_the_institute_for_global_prosperity_.pdf

54 von Parijs, P., & Vanderborght, Y. (2017). *Basic income: A radical proposal for a free society and a sane economy*. Cambridge, MA: Harvard University Press.

55 A small‐scale model for such accounts can be found in the ‘points‐time‐accounts’ in St. Gallen in Switzerland. For more information, with links to feasibility studies etc., see Otto, U. (2013). ‘Zeitvorsorge’‐Modelle, Seniorengenossenschaften & Co. *Institut für Soziale Arbeit der FHS St. Gallen*. Available at: http://www.ifsa.ch/3472 [Accessed July 11, 2017]; Stadt St. Gallen (2017). *Zeitvorsorge*. Retrieved July 11, 2017, from https://www.stadt.sg.ch/home/gesellschaft‐sicherheit/aeltere‐menschen/zeitvorsorge.html

56 For example, substance abuse framed as a criminal activity rather than a problem of social deprivation suggests completely different strategies to address it. Schön, D. A., & Rein, M. (1995). *Frame reflection. Towards the resolution of intractrable policy controversies*. New York, NY: Basic Books; Lakoff, G. (2004). *Don’t think of an elephant. Know your values and frame the debate*. Hartford, VT: Chelsea Green Publishing.

